# Haploidy in Tobacco Induced by *PsASGR-BBML* Transgenes via Parthenogenesis

**DOI:** 10.3390/genes11091072

**Published:** 2020-09-12

**Authors:** Zhifen Zhang, Joann Conner, Yinping Guo, Peggy Ozias-Akins

**Affiliations:** Department of Horticulture and Institute of Plant Breeding, Genetics & Genomics, University of Georgia, Tifton, GA 31793, USA; zhifen@uga.edu (Z.Z.); jconner@uga.edu (J.C.); yguo@uga.edu (Y.G.)

**Keywords:** apomixis, parthenogenesis, haploid progeny, dicotyledon, *PsASGR-BBML*, pseudogamy

## Abstract

Background: Engineering apomixis in sexually reproducing plants has been long desired because of the potential to fix hybrid vigor. Validating the functionality of genes originated from apomictic species that contribute to apomixis upon transfer to sexually reproducing species is an important step. The *PsASGR-BABYBOOM-like* (*PsASGR-BBML*) gene from *Pennisetum squamulatum* confers parthenogenesis in this apomict, and its functionality was demonstrated in several sexually reproducing monocots but not in any dicots. Methods: We introduced the *PsASGR-BBML* gene regulated by egg cell-specific promoters, either *AtDD45* or *AtRKD2*, into tobacco, and analyzed progeny of the transgenic lines resulting from self-pollination and crossing by flow cytometry. Results: We identified haploid progeny at a frequency lower than 1% in the *AtDD45_pro_* lines, while at a frequency of 9.3% for an octoploid (2*n* = 8*x*) *AtRKD2_pro_* line. Haploid production in the T_2_ generation, derived from the tetraploid T_1_ offspring of this original octoploid *AtRKD2_pro_* line, was also observed. Pollinated by homozygous transgenic tobacco carrying a *DsRed* marker gene, 4*x* progeny of the *AtRKD2_pro_* line yielded parthenogenetic embryos identified as DsRed negative. We verified that the DsRed negative seedlings recovered were haploid (2*x*). Conclusion: The *PsASGR-BBML* gene regulated by egg cell-specific promoters could enable parthenogenesis in tobacco, a dicotyledon species.

## 1. Introduction

Flowering plants (angiosperms) can reproduce both sexually and asexually. In female sexual reproduction, a nucellar cell goes through meiosis to produce four megaspores, one of which divides by mitosis to develop into the female gametophyte (embryo sac) containing an egg cell with two flanking synergid cells at the micropylar end, a central cell with two polar nuclei, and antipodal cells at the chalazal end. Microspore mother cells in anthers go through meiotic division and develop into the male gametophytes (pollen grains). The haploid gametophytes complete the life cycle by developing gametes which fuse to initiate the diploid sporophyte. Angiosperms require the process of double fertilization; the egg cell (1*n*) merges with a sperm cell (1*n*) to form a zygote (2*n*) that develops into an embryo encapsulated within a seed, while the central cell (2*n*) fuses with the second sperm cell (1*n*) from the same pollen grain to initiate endosperm (3*n*) development. In dicots, the endosperm typically is absorbed during seed maturation, while in monocots it provides nutrition for germinating seeds [1]. In contrast, as an asexual reproduction pathway, apomixis produces seeds without double fertilization. The mechanisms of apomixis can be complex and diverse across species and have been reviewed intensively [2,3,4,5]. In general, various forms of gametophytic apomixis share three common components: (1) apomeiosis by which unreduced female gametophytes form; (2) parthenogenesis by which female gametes develop into embryos without fertilization; (3) formation of endosperm autonomously or following fertilization of the central cell by a sperm cell (pseudogamy). If parthenogenesis occurs independently from apomeiosis, haploid progeny can be obtained when viable endosperm develops autonomously or after the central cell is fertilized.

Parthenogenesis is genetically controlled as reviewed previously [6]. As a member of the *AP2*-domain transcription factor superfamily, a *PsASGR-BABYBOOM*-like (*PsASGR-BBML*) gene isolated from an apomictic grass, *Pennisetum squamulatum*, within the Apospory-Specific Genomic Region (ASGR) [7], was the first gene shown to confer parthenogenesis in plants with its functionality validated in sexual pearl millet (*P. glaucum*) [8]. The promoter of the *PsASGR-BBML* gene was found to control gene expression restricted to the egg apparatus (egg/synergid complex) of the unfertilized embryo sac [8], indicating that egg-specific expression of the *PsASGR-BBML* gene could be critical for the resulting parthenogenesis phenotype. Later, the functionality of the *PsASGR-BBML* gene regulated by either its native promoter or an egg cell-specific promoter *DD45* (*DOWN REGULATED IN DETERMINANT INFERTILE* (*DD*) 45) from *Arabidopsis* [9] was validated in two additional monocot species, rice (*Oryza sativa*) and maize (*Zea mays*) [10]. However, when the *PsASGR-BBML* gene regulated by the *At*DD45 promoter was introduced into *Arabidopsis*, no significant haploid production was observed, even though the gene was expressed within ovules and no splicing changes were identified [10]. The exact reason why the *PsASGR-BBML* gene failed to enable parthenogenesis in *Arabidopsis*, a dicot species, remains unknown.

Given its amphiploid origin [11], tobacco (*Nicotiana tabacum*; 2*n* = 4*x* = 48) can provide an alternative scenario to test the functionality of the *PsASGR-BBML* gene in a dicot species, where the gene will express in a di-haploid egg cell. The recovery/survival of haploid progeny was reported at a very low frequency in tobacco [12,13,14]. When seeds resulted from self-pollination, only one individual of a surviving twin was identified as haploid out of 104,000 germinated seeds representing 12 varieties, indicating a frequency of approximately one in 10,000 or lower [12]. When seeds resulted from intraspecific crosses, haploid progeny were identified with the aid of genetic markers at a frequency ranging from 0.02% to 0.2% [12,13,14], which varied depending on the combinations of selected parents. Our objective was to test whether introducing the *PsASGR-BBML* gene could significantly increase the tendency of haploid production in amphiploid tobacco. In order to test the functionality of the *PsASGR-BBML* gene in tobacco, eudicot egg cell-specific promoters are preferred to regulate the transgene, as the specific expression of the *PsASGR-BBML* gene in the egg cell was considered essential for the parthenogenesis phenotype and its endogenous promoter was not functional in dicots [8,10]. Over-expression of the *PsASGR-BBML* by *CaMV35S* (*Cauliflower Mosaic Virus 35S*) promoter in *Arabidopsis* led to the formation of somatic embryo-like structures on the adaxial leaf surface and ectopic shoot/leaf development [15] rather than parthenogenesis, comparable to the observation in transgenic *Arabidopsis* and *Brassica* where the *BnBBM1* and *BnBBM2* genes were over-expressed [16]. The promoters of *AtRKD1* and *AtRKD2*, members of a class of transcription factors containing a *RWP-RK domain* (*RKD*) that are important to maintain the egg identity in *Arabidopsis*, drove gene expression specifically in egg cells [17] so that they provide alternatives to the *AtDD45* promoter to regulate the *PsASGR-BBML* gene in dicot plants.

The study herein reported was an effort to test the functionality of the *PsASGR-BBML* transgene regulated by two egg cell-specific promoters in tobacco and validate whether this gene isolated from a monocot apomict could lead to haploid production via parthenogenesis in a dicot species.

## 2. Materials and Methods

### 2.1. Vector Construction

Two cassettes of the genomic *PsASGR-BBML* sequence (EU559280) fused with egg cell-specific promoters were generated. The cassette *AtDD45_pro_:gPsASGR-BBML* contained a 1008 bp promoter of *Arabidopsis DD45/EC1.2* (At2g21740) [9], the 3540 bp of the *PsASGR-BBML* gene from BAC p208 [18,19], including 8 exons and 7 introns, and 609 bp 3′ of the stop codon plus the predicted poly(A) signal. The cassette was subsequently ligated into pCambia1300 (CAMBIA, Canberra, Australia) for hygromycin selection in transgenic plants [10]. The recombinant vector pCambia1300-*AtDD45_pro_:gPsASGR-BBML* (ddBR1) was introduced into *Agrobacterium* strains AGL1 and LBA4404 for plant transformation.

The 521 bp promoter region of *AtRKD2* (At1g74480), previously verified as egg-cell expressing in Arabidopsis, [17] was amplified from genomic DNA of *Arabidopsis* Columbia using the primer combination p3905/3906 (Table S1). The sequence of the *AtRDK2* promoter was verified. The *AtDD45* promoter in the cassette *AtDD45_pro_:gPsASGR-BBML* was replaced with the *AtRKD2* promoter and the new cassette *AtRKD2_pro_:gPsASGR-BBML* was ligated into pCambia2300 for kanamycin selection in transgenic plants. Kanamycin selection offered a simpler scheme to separate transformant T1 progeny from non-transformant T1, compared with the use of hygromycin selection (see Section 2.5). The new recombinant vector pCambia2300-*AtRKD2_pro_:gPsASGR-BBML* (*RKD2gBB*) was verified by additional restriction mapping, and introduced into *Agrobacterium* strain EHA105, as AGL1 led to relatively severe tissue browning during the transformation of ddBR1.

### 2.2. Plant Transformation

Two vectors were introduced into tobacco by *Agrobacterium*-mediated transformation adapted from Clemente [20]. Leaves from 1-month-old tobacco plants (PI 552484, *N. tabacum* cv. Xanthi NN, seeds purchased from Lehle Seeds, Round Rock, TX, USA) grown in the greenhouse were collected, immersed in reverse osmosis deionized (RODI) water, and then surface sterilized with 10% commercial bleach (Clorox, Oakland, CA, USA, (6% sodium hypochlorite)) for 10 min, followed by 5× rinse with sterile RODI water. After removal of the midribs, the leaves were cut into 1–2 cm^2^ explants and cultured on a shoot induction medium (SIM) consisting of Murashige and Skoog salts and vitamins (M519, PhytoTechnology Laboratories, Lenexa, KS, USA), 3% (*w*/*v*) sucrose (Research Products International, Mt Prospect, IL, USA), 1 mg/L 6-benzylaminopurine (BA), 0.1 mg/L 1-naphthaleneacetic acid (NAA), and 8 g/l agar with pH adjusted to 5.7. After 2 days of culture, the leaf disc explants were inoculated with *Agrobacterium* by immersing the explants in *Agrobacterium* suspension (OD_600_ = 0.5~0.6, in liquid SIM containing 200 µM acetosyringone and 0.02% (*v*/*v*) Silwet-77 (Lehle Seeds, Round Rock, TX, USA)) for 15~20 min. After blotting dry with sterile filter paper, explants were placed on fresh SIM for co-culture. After 2 days of co-culture, explants were transferred to SIM containing 30 mg/L meropenem (ABBLIS Chemicals, Houston, TX, USA), plus 30 mg/L hygromycin or 200 mg/L kanamycin for ddBR1 and RKD2gBB, respectively. Each plate contained 5–7 explants which were transferred to fresh SIM with antibiotic selection every 2 weeks. When shoots reached 2–3 cm, they were excised and transferred to a root induction medium (RIM) containing MS salts and vitamins, 3% (*w*/*v*) sucrose, 0.1 mg/L NAA, and 8 g/L agar with pH adjusted to 5.7, plus 30 mg/L meropenem and the same selective agent used in SIM. Rooted plantlets were transferred to the Magenta™ GA7 vessels (Sigma-Aldrich, St. Louis, MO, USA), containing 0MS medium (SIM without plant growth regulators) with 30 mg/L meropenem and the same selective agent used in SIM, for further development. Plantlets derived from different leaf disc explants were considered independent transformation events while those from the same explants were considered potentially the same lines. Plantlets of 5–8 cm height were transferred to soil for acclimation. After genotyping, at least two plants from each independent line with a full-length *PsASGR-BBML* transgene were grown in the greenhouse to set seeds. All tissue cultures were maintained at 26 °C with a 16/8 h light cycle. Unless otherwise noted, all chemicals were from Sigma-Aldrich (St. Louis, MO, USA).

### 2.3. Genotyping

Genomic DNA was isolated from T0 transgenic plants and their progeny by using the CTAB method [21]. The presence of the full-length *gPsASGR-BBML* transgene in the T0 transgenic lines and the haploid T1 progeny was verified by polymerase chain reaction (PCR) using the primer combination p1792/1801 (Table S1) to amplify the region from the start of the open reading frame (ORF) through 159 bp of the 3′ UTR. PCR reactions consisted of 1× PrimeSTAR GXL Buffer, 200 µM dNTP, 0.2 µM primers, 0.625 U PrimeSTAR GXL DNA Polymerase (Takara Bio USA, Inc. Mountain View, CA, USA), and 50–100 ng genomic DNA in a 25 µL reaction followed by 35 cycles of amplification with a cycling condition of 15 s at 98 °C, 15 s at 60 °C, and 4 min at 68 °C. In order to confirm the presence of the transgenes in the haploid T2 progeny of line RKD2gBB, progeny were genotyped by PCR using primer combinations p3767/4127 and p4303/4304 (Table S1). PCR reactions consisted of 1× GoTaq^®^ Master Mix (M7123, Promega, Madison, WI) and 50–100 ng genomic DNA in a 20 µL reaction followed by 32 cycles of amplification with a cycling condition of 15 s at 95 °C, 15 s at 60 °C, and 30 s at 72 °C. PCR products were visualized under UV light after electrophoresis in 1% agarose gels and staining with ethidium bromide.

### 2.4. Estimation of Transgene Copy Numbers by Quantitative PCR

In order to estimate the copy number of the transgenes in T0 and T1 plants, genomic DNA from transgenic tobacco was digested overnight with *Eco*RI-HF^®^ (NEB, Ipswich, MA, USA), followed by purification using the DNA Clean and Concentrator Kit (D4033, Zymo Research, Irvine, CA, USA). The purified DNA, after dilution 20-fold, was used as a template for quantitative PCR (qPCR) using a LightCycler^®^ 480 system (Roche, Basel, Switzerland). PCR reactions were set up following the manufacturer’s instruction of the LightCycler^®^ 480 SYBR Green I Master mix V13 (Roche) and conducted using the SYBR green I/HRM dye program with the primer combination p4303/4304 for the *PsASGR-BBML* transgene and the primer combination p4133/4134 for the *tubulin* gene (NCBI Reference Sequence: XM_016623993) as a reference gene [22]. The amplification efficiency of each assay was estimated based on the qPCR data of a 5-log serial dilution (0.0016, 0.008, 0.04, 0.2, 1×) of a DNA mixture that contained an equal amount of DNA from each tested sample by using the absolute quantification method in the software of the Lightcycler^®^ 480 system. Each reaction had two technical replicates. The qPCR data were analyzed by using the advanced relative quantification method in the software of the Lightcycler^®^ 480 system to estimate the copy numbers of the *PsASGR-BBML* transgene in transgenic tobacco plants.

### 2.5. Seed Germination Assay for Transgenic Progeny

Seeds were surfaced sterilized using a 16-h chlorine gas treatment (2 mL 12 M HCl into 200 mL commercial bleach in a 10 L desiccator) or immersing in 10% commercial bleach (Clorox) for 10 min followed by 5× rinse with sterile RODI water. Since cotyledons could turn green despite the lack of transgene when seeds were germinated on hygromycin-containing medium and unreduced transgenic progeny potentially could outcompete the haploid plants with prolonged culture on hygromycin-containing medium, a selection scheme was developed to quickly separate unreduced transgenic progeny from their haploid siblings and non-transgenic with a short culture period on hygromycin-containing medium, based on a method developed for *Arabidopsis* [23]. Seeds of the ddBR1 lines were sown on 0MS medium containing 60 mg/L hygromycin and incubated in the dark for 7–10 days. Seedlings displaying elongated hypocotyls (>0.5 cm, mostly 0.8~1 cm) were considered to be transgenic while seedlings that did not elongate (<0.5 cm, mostly 0.1~0.3 cm) were considered to be either non-transgenic or potentially haploid transgenic. After transfer to 0MS for further growth with a 16/8 h light cycle, short seedlings that turned green were considered potential transgenic haploids while seedlings that failed to turn green and grow further were considered to be non-transgenic. The ploidy levels of those potentially haploid transgenic plants were examined by flow cytometry within a month. Adapted from [24], seeds of the RKD2gBB lines were sown on 0MS containing 600 mg/L kanamycin which was intended to prevent non-transgenic escape. After a 2-week culture period, green seedlings were considered to be transgenic while seedlings showing chlorosis (i.e., fully or partially whitening) at cotyledons or the shoot apex were considered to be non-transgenic. Those resistant transgenic seedlings were transferred to 0MS for further growth and their ploidy levels were examined by flow cytometry within a month. In order to estimate the baseline of autonomous haploid production of the PI 552484, non-transgenic seeds were sown on 0MS and the seedlings that did not elongate after 7 days in the dark were considered to be potentially haploid. Those small seedlings were transferred to fresh 0MS for further growth with a 16/8 h light cycle, and their ploidy levels were examined by flow cytometry within a month.

### 2.6. Flow Cytometry

Possible parthenogenesis in T0 transgenic plants was first determined by flow cytometric seed screen (FCSS) [25]. Preliminary microscopy study found most of the endosperm was absorbed by the embryo when capsules started to turn brown but were not completely desiccated (21–28 days after pollination). Capsules at this developmental stage were selected for FCSS as seeds were not too hard to chop while the flow cytometric signal would be mostly from the developing embryos rather than the endosperm. Bulked seed samples from two individual capsules were processed for each transgenic plant adapted from Conner [8]. In brief, 50–100 developing seeds together with young leaf tissue of sorghum (*Sorghum bicolor*) or cowpea (*Vigna unguiculata*) as a genome-size standard were chopped in 200 µL LB01 lysis buffer consisting of 15 mM Tris, 2 mM Na_2_EDTA, 0.5 mM spermine tetrahydrochloride, 80 mM KCl, 20 mM NaCl, 0.1% (*v*/*v*) Triton X−100, pH 7.5 and 16 mM 2-mercaptoethanol, and filtered with a 30 µm CellTrics disposable filter (Sysmex Partec, Görlitz, Germany). After propidium iodide solution containing RNase (Cat# 550825, BD Biosciences, San Jose, CA, USA) was added at half volume, the filtered samples were incubated on ice for at least 15 min, followed by analysis using a BD Accuri C6 flow cytometer (BD Biosciences) with gating set by the selection of objects with a strong correlation between FL2 and FL3 signals using a flow rate of 35 µL sample per min. Events were collected within the gated region for each sample.

In order to identify haploid seedlings, selected tobacco seedlings (see Section 2.5) were analyzed by flow cytometry when they reached at least the 4-leaf stage. Leaf tissues (1 mm^2^ or less) from five seedlings were pooled together and processed as described previously by flow cytometry. If a bulk sample showed a haploid signal, leaf tissue from each individual comprising the bulk was collected and processed by flow cytometry to identify the haploid individuals. The efficiency of haploid production was determined based on the percentage of haploid seedlings among the total seedlings germinated.

### 2.7. Transcription Analysis of the PsASGR-BBML Transgene in Tobacco Ovules

Ovules were isolated from 6–7 flowers at stage 11 of tobacco flower development [26] and stored in the RNA*later*™ Storage Solution (Invitrogen, Carlsbad, CA, USA). Total RNA was extracted using the RNeasy^®^ Plant Mini Kit (QIAGEN, Hilden, Germany), followed by a DNase (Cat# 18068015, ThermoFisher Scientific, Waltham, MA, USA) treatment according to the manufacturer’s recommendation to remove genomic DNA. Single strand cDNA was synthesized with the SuperScript III First-strand Synthesis System (Invitrogen) by reverse transcription PCR (RT-PCR). The presence of the *PsASGR-BBML* transcript including 8 exons was verified by PCR using the primer combination p1792/1793 (Table S1). PCR reactions consisted of 2 µL of the first-strand cDNA synthesis reaction, 1× PrimeSTAR GXL Buffer, 200 µM dNTP, 0.2 µM primers, 0.625 U PrimeSTAR GXL DNA Polymerase in a 25 µL reaction (Takara Bio USA, Inc.) followed by 35 cycles of amplification with a cycling condition of 15 s at 98 °C, 15 s at 60 °C, and 2 min at 68 °C. PCR products were visualized under UV light after electrophoresis in 1% agarose gels and staining with ethidium bromide. 

### 2.8. Sequence Analysis of the PsASGR-BBML Transgene from Line RKD2gBB_6.1

Total RNA from ovules of flowers at stage 11 from two haploid-producing 4*x* T1 progeny (K6.1_20 and K6.1_23) of line RKD2gBB_6.1 was extracted, DNase treated and converted into single strand cDNA as previously stated. PCR reactions consisted of 2 µL of the first-strand cDNA synthesis reaction, 1× PrimeSTAR GXL Buffer, 200 µM dNTP, 0.2 µM primers, and 0.625 U PrimeSTAR GXL DNA Polymerase in a 25 µL reaction (Takara Bio USA, Inc) followed by 35 cycles of amplification with a cycling condition of 15 s at 98 °C, 15 s at 60 °C, and 2 min at 68 °C. Primers p1792/1801 (Table S1) were used to amplify the transcript from the start of the ORF through to the 3′ UTR. Amplified PCR products from each sample were directly cloned into PCR4-TOPO (Invitrogen) vector with cloned inserts sequenced at Psomagen (Rockville, MD, USA). Sequencing data were analyzed using Geneious prime software (Biomatters Limited, Auckland, New Zealand).

### 2.9. Tobacco Crossing

Flowers were emasculated at stage 10 [26] in the evening before anthesis. The emasculated flowers were pollinated the following morning, tagged and capped with tailored pollination envelopes. The 4*x* T1 plants of line RKD2gBB_6.1 (an octoploid, 2*n* = 8*x*) were pollinated by non-transgenic tobacco. The 4*x* T2 plants of line RKD2gBB_6.1 were pollinated by homozygous T3 plants of a transgenic tobacco line (2*n* = 4*x*) carrying a cassette of a *DsRed* gene regulated by the promoter of soybean (*Glycine max*) *elongation factor 1a* (*GmEF1a*, Glyma.17G186600, Phytozome v12) and the *nopaline synthase* (*NOS*) terminator that segregated as a single locus (Zhang unpublished). The embryos and seedlings without DsRed fluorescence were considered to be parthenogenetic, since these progenies did not inherit the *DsRed* transgene from the male parent.

### 2.10. Embryo Isolation and Observation

Capsules were harvested 9 through 17 days after pollination. Ovules were isolated and macerated in an enzymatic solution [27] composed of 12% (*w*/*v*) mannitol, 3 mM MES, 1% (*w*/*v*) cellulose R-10 (Research Products International), and 0.8% (*w*/*v*) *Rhizopus sp.* pectinase, with pH adjusted to 5.7, for 30 min with agitation at 60 rpm. The ovules were rinsed in a washing buffer composed of 12% (*w*/*v*) mannitol and 3 mM MES (pH 5.7) at least 3 times and then gently ground with a small pestle to release the developing embryos. Without removing integument debris, isolated embryos were mounted on a glass slide in the washing buffer and observed under a microscope (Zeiss, Thornwood, NY, USA) equipped with a PhotoFluor LM-75 illuminator (89 North, Williston, VT, USA) and a Ds-RED filter (excitation: 545/25 nm, emission: 605/70 nm, Chroma Technology, Bellows Falls, VT, USA). Images were taken using an AxioCam camera (Carl Zeiss, Oberkochen, Germany) and the AxioVision LE64 software. Embryos without visible damage were counted. Unless otherwise noted, all chemicals were obtained from Sigma-Aldrich.

## 3. Results

### 3.1. Recovery of Transgenic Tobacco 

Thirty and seventeen T0 lines that contained all eight exons, seven introns and 3′UTR were recovered, respectively, for the cassettes *AtDD45_pro_:gPsASGR-BBML* (ddBR1) and *AtRKD2_pro_:gPsASGR-BBML* (RKD2gBB). In general, all transgenic lines displayed wild type vegetative growth, except for one ddBR1 line that was severely stunted. Upon entering the reproductive stage, most T0 lines showed normal flower development and produced seeds by self-pollination. In some T0 lines, short stamens and poor pollen production were observed. By hand-pollination with their own pollen when available, all lines with abnormal phenotypes produced viable seeds except for the severely stunted ddBR1 line. The *PsASGR-BBML* transcript including eight exons was detected in ovules of ten transgenic lines that were randomly selected for each construct at stage 11 of tobacco flower development (Table S2).

### 3.2. Haploid Production of T0 Transgenic Tobacco

As tobacco is an allotetraploid (2*n* = 4*x*), progeny were considered “haploid” when their genome size was calculated at half of their maternal parent. Five independent ddBR1 lines (5/29, 17.2%) and three independent RKD2gBB lines (3/17, 17.6%) displayed a haploid (2*x* since tobacco is tetraploid) signal when immature seeds were analyzed by FCSS (Figure S1). All T0 transgenic lines remained tetraploid (2*n* = 4*x*) except for three RKD2gBB lines (RKD2gBB_4.1, RKD2gBB_6.1 and RKD2gBB_7.1) which became octoploid (2*n* = 8*x*), probably due to genome duplication during tissue culture. According to FCSS, two of the haploid-producing RKD2gBB T0 lines (RKD2gBB_6.1 and RKD2gBB_7.1) were octoploid (2*n* = 8*x*, Figure S1). The ploidy levels of the haploid-producing lines were confirmed by flow cytometry analysis using leaf tissue of the T0 plants, and these haploid-producing lines were analyzed in further detail.

**Figure 1:**
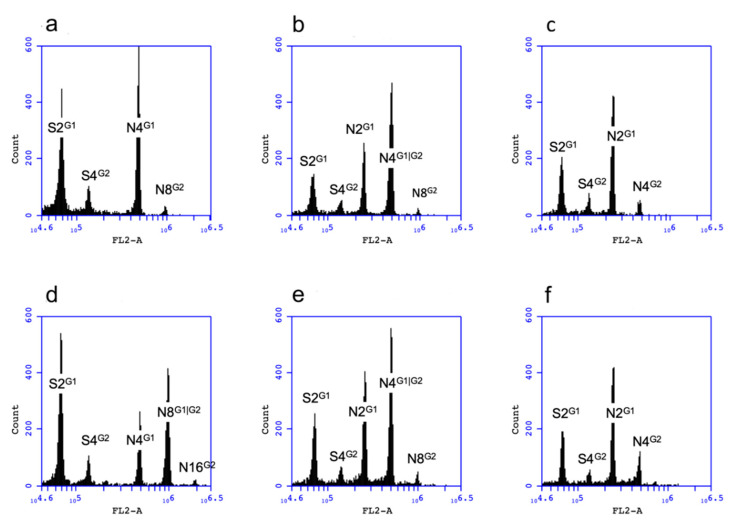
Representative flow cytometry analysis. (**a**) A bulked sample of five non-transgenic tobacco plants; (**b**) a bulked sample of T1 progeny of a haploid-producing 4*x* ddBR1 line (ddBR1_240.2) with 2*x* signal; (**c**) a 2*x* T1 progeny of line ddBR1_240.2; (**d**) a bulked sample of T1 progeny of haploid-producing 8*x* line RKD2gBB_6.1 with 4*x* signal; (**e**) a bulked sample of T2 progeny of a 4*x* T1 plant (K6.1_20) of line RKD2gBB_6.1 with 2*x* signal; (**f**) a 2*x* T2 progeny of a 4*x* T1 plant K6.1_20. S2^G1^ and S4^G2^ designate 2*n*/2*x*/2c and 2*n*/2*x*/4c peaks of sorghum or cowpea, N2^G1^, N4^G1^, N4^G2^, N4^G1|G2^, N8^G2^, N8^G1|G2^, and N16^G2^ designate 2*n*/2*x*/2c, 2*n*/4*x*/4c, 2*n*/2*x*/4c, 2*n*/4*x*/4c|2*n*/2*x*/4c, 2*n*/4*x*/8c, 2*n*/8*x*/8c|2*n*/4*x*/8c, and 2*n*/8*x*/16c peaks of tobacco, respectively.

In a preliminary study, T1 seedlings of a haploid-producing ddBR1 line that germinated on 0MS containing 60 mg/L hygromycin and elongated after seven days of culture in the dark were all tetraploid (4*x*) as their T0 parent. Haploid (2*x*) seedlings were only identified among seedlings that failed to elongate during the first seven days on 0MS containing hygromycin. Thus, we decided to focus on the T1 seedlings of the ddBR1 lines that failed to elongate on 0MS containing 60 mg/L hygromycin within 10 days where haploid plants would be more likely to be identified. Haploid (2*x*) seedlings were identified among T1 seedlings of three haploid-producing ddBR1 lines (2*n* = 4*x*), ddBR1_204.2, ddBR1_205.1 and ddBR1_240.2, at a frequency lower than 1%, while no haploid T1 seedlings were recovered from the other two haploid-producing ddBR1 lines, ddBR1_13.1 and ddBR1_204.1, showing FCSS haploid peaks (Table 1, Figure 1). This could be due to the frequency of haploid seedling production against the number of seedlings screened. In contrast, among the three haploid-producing RKD2gBB lines, haploid (4*x*) T1 seedlings were identified from one octoploid (2*n* = 8*x*) line (RKD2gBB_6.1) at a frequency of 9.3% while no haploid plant was identified in the other two lines, RKD2gBB_3.2 and RKD2gBB_7.1, showing FCSS haploid peaks (Table 1, Figure 1). In comparison, 397 seedlings that remained stunted within the first two weeks of growth on 0MS from 3700 non-transgenic seedlings were analyzed by flow cytometry, and no haploid seedlings were identified (Figure 1).

All haploid-producing lines had a relatively simple integration of the transgene. Based on the elongation of T1 seedlings germinated in the dark, indicating resistance to hygromycin, the transgene likely segregated as a single locus in all five haploid-producing T0 ddBR1 lines except for line ddBR1_204.1 showing segregation distortion of the transgene and having an inflated number of T1 seedlings characterized as “susceptible” (Table S3). Among the haploid-producing RKD2gBB lines, the transgene segregated as a single locus in lines RKD2gBB_3.2 and RKD2gBB_7.1, and as two loci in the line RKD2gBB_6.1 which showed the highest efficiency in haploid production (Table S3). When genotyped by PCR, all haploid T1 plants identified carried the full-length sequence of *PsASGR-BBML* transgene. Quantitative PCR further suggested that the haploid-producing T0 lines should have one or two copies of the transgene integrated except for one line (Table S3).

### 3.3. Haploid Production of the Progeny Derived from Line RKD2gBB_6.1

As tobacco is an allotetraploid, haploid T1 plants derived from the ddBR1 lines could not form normal embryo sacs due to the lack of homologous chromosomes for pairing at meiosis. As a result, they were sterile and produced no seeds. Since line RKD2gBB_6.1 was 8*x*, its haploid (4*x*) T1 were fertile and capable of producing seeds, providing an opportunity to verify the functionality of the *PsASGR-BBML* transgene among progeny lines even when the ploidy level reduced by half in the second generation. Similar to the T0 generation, T1 plants of line RKD2gBB_6.1 tended to have short stamens and many empty anthers. Nevertheless, seeds could still be produced by hand-pollination with their own pollen as long as some pollen grains were available. Among nine 4*x* T1 plants derived from line RKD2gBB_6.1 (2*n* = 8*x*), three were able to produce T2 seeds after hand-pollination. The other six plants produced no T2 seeds after attempted hand-pollination as functional pollen grains were not produced from these plants. Haploid (2*x* and 4*x*) T2 seedlings were identified from the three 4*x* T1 plants (K6.1_11, K6.1_20 and K6.1_23) and four 8*x* T1 plants (K6.1_1, K6.1_12, K6.1_17, and K6.1_22) analyzed, respectively (Table 2, Figure 1). The efficiency of haploid production ranged from 2.7% to 27.3%, showing no clear correlation to the numbers of loci inherited or the copy numbers of the transgene (Table 2 and Table S4). The T1 plants that inherited one locus or both were able to produce haploids (Table 2 and Table S4). In general, the efficiency of haploid production tended to be higher in the 8*x* T1 than the 4*x* T1. The 8*x* T1 plants tended to set seeds more poorly with higher frequencies of haploid progeny associated with poorer seed setting and where fewer seeds were available for analysis (Table 2). 

Haploid (2*x*) progeny were obtained at a frequency of 5.6% when a 4*x* T1 plant of line RKD2gBB_6.1 was pollinated by non-transgenic tobacco (Table 3). The number of seedlings susceptible to kanamycin inflated in the F1 to about three times as many as the resistant seedlings (Table S5). Segregation distortion was also observed in progeny from the 4*x* T1 plants of line RKD2gBB_6.1 that inherited one or both transgene loci. The number of resistant seedlings was much lower than expected when these T1 plants were self-pollinated and a relatively large proportion of seeds failed to germinate (Table S4). When genotyped, all haploid T2 progeny identified carried the *PsASGR-BBML* transgene and the *nptII* marker gene. Attempts to recover homozygous T1 plants of any haploid-producing ddBR1 lines were unsuccessful. A homozygous T1 plant of line RKD2gBB_6.1 was likely recovered (K6.1_7, Table S4) according to the copy number estimated by qPCR, but it only produced distorted flowers and no seeds.

### 3.4. Transcription of the PsASGR-BBML Transgene in the 4x T1 of Line RKD2gBB_6.1

The presence of the *PsASGR-BBML* transcript including eight exons was verified in all four 4*x* T1 plants of line RKD2gBB_6.1 (Figure S2) along with four T1 plants not displaying parthenogenesis. Sequencing data showed that the *PsASGR-BBML* transcript from two 4*x* T1 plants (K6.1_20 and K6.1_23) of line RKD2gBB_6.1 was identical to the original transcript, showing no alternative splicing or sequence alteration to explain the higher rate of parthenogenesis in this line.

### 3.5. Observation of Parthenogenetic Embryos

**Figure 2:**
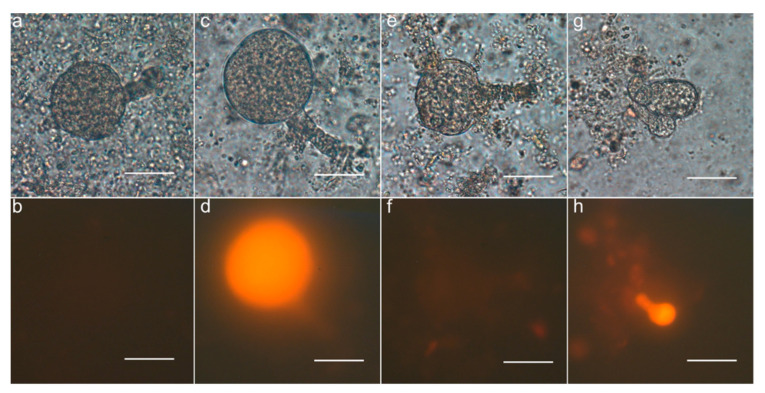
Expression of DsRed in developing embryos of the progeny from crosses with a homozygous transgenic tobacco line carrying a DsRed marker gene. (**a**,**c**,**e**,**g**) Bright field; (**b**,**d**,**f**,**h**) Ds-RED filter. (**a**,**b**) Progeny of the non-transgenic obtained by self-pollination. (**c**,**d**) Progeny of the non-transgenic crossed with the homozygous DsRed transgenic plant; (**e**,**f**) DsRed negative progeny of K6.1_12_26 crossed with the homozygous DsRed transgenic plant; (**g**,**h**) DsRed positive and negative progeny of K6.1_17_7 crossed with the homozygous DsRed transgenic plant. Bar = 50 µm.

Attempts to observe parthenogenetic embryos via ovule clearing from emasculated flowers of the haploid-producing lines were unsuccessful. This method had been used to successfully identify parthenogenesis in pearl millet transgenic lines [8]. Tobacco flowers abscised approximately six days after emasculation with no defined embryos observed in cleared ovules. Pollination was needed to prevent flower abscission, and to ensure that haploid embryos could further develop and be identified. In preliminary studies to enzymatically isolate embryos from the several hundred ovules of a capsule, only a small number of four-celled embryos were observed seven days after pollination. This suggested that either tobacco embryos at the four-cell stage were too fragile to isolate or that the majority of embryos might be at an earlier developmental stage that could be easier to damage during isolation or harder to identify after isolation. We decided to examine capsules at least nine days after pollination when the majority of embryos entered the eight-cell stage or later. After crossing with pollen from tobacco plants homozygous for a DsRed fluorescence marker gene driven by a strong constitutive promoter GmEF1apro, 4*x* T2 progeny derived from three 8*x* T1 plants of line RKD2gBB_6.1 that inherited one or both loci of the transgene were able to produce embryos or seeds without DsRed fluorescence (Figure 2, Table 4). The DsRed negative embryos were considered to be parthenogenetic due to the lack of the DsRed transgene transmitted by the paternal parent. Developing parthenogenetic embryos without DsRed fluorescence were observed at a frequency from 3.2% to 8.0% among different 4*x* T2 plants of line RKD2gBB_6.1 (Table 4) which was comparable to the frequency of haploid production observed when a 4*x* T1 plant was pollinated by wildtype pollen (Table 3). Those DsRed negative parthenogenetic embryos tended to develop as normally as the DsRed positive embryos from the same capsule, though abnormality (e.g., slow-growth, deformity, etc.) was occasionally observed in some of the DsRed negative embryos. Seedlings without DsRed fluorescence were also identified when the mature seeds from capsules obtained by crossing were germinated, and they were verified as haploid (2*x*) by flow cytometry (Table 5). According to PCR genotyping, these 2*x* haploid seedlings carried the *PsASGR-BBML* transgene but not the DsRed gene.

## 4. Discussion

This report demonstrates that the *PsASGR-BBML* transgene, regulated by egg cell-specific promoters, enabled tobacco to produce haploid progeny via parthenogenesis as previously observed in sexual pearl millet [8], rice and maize [10]. While haploid production by self-pollination has been reported in some tobacco varieties at a frequency of approximately 0.01% [12], the haploid frequency observed in the current study with transgenic tobacco was 20 to 900 times higher. In addition, haploid progeny was not identified among seeds resulting from self-pollination of the non-transgenic PI line used to produce the transgenic lines. This indicates that haploid production was a gain of function attributable to the transgene rather than a propensity caused by the genetic composition of the PI. Expression of *PsASGR-BBML* in egg cells presumably transforms the egg cells into a zygote-like state, inducing embryogenesis from unfertilized eggs. Use of an egg cell-specific promoter *AtDD45* to regulate the gene *PsASGR-BBML* or *OsBBM1* whose protein sequence clustered with the PsASGR-BBML protein in a phylogenetic subclade [8] was able to induce parthenogenetic embryos in rice [10,28]. Compared with the monocot species tested [10], the efficiency of haploid production in transgenic tobacco where the *PsASGR-BBML* transgene was regulated by the *AtDD45* promoter was low (Table 1). Given that embryogenesis in monocots significantly diverges from dicots [29], the *PsASGR-BBML* transgene by itself may be inefficient, if sufficient, to transform the egg cell into a zygote-like cell in tobacco, which was also implied in the study of *Arabidopsis* where haploid progeny were not recovered from any transgenic lines expressing the *PsASGR-BBML* transgenes [10]. The reasons why the *PsASGR-BBML* transgene functioned more efficiently in tobacco than *Arabidopsis* remains unknown.

The higher efficiency of haploid production in the RKD2gBB line than the ddBR1 lines was likely attributable to temporal or spatial differences in the promoter activity. When haploid progeny were identified, the RKD2gBB line (RKD2gBB_6.1) yielded haploid progeny at a frequency at least 10 times higher than the ddBR1 lines (Table 1). While *AtDD45_pro_* and *AtRKD2_pro_* were both considered to be egg cell-specific, gene expression regulated by the *AtRKD2_pro_* was tightly restricted to the egg cell [17] while the expression regulated by the *AtDD45_pro_* was observed in the egg cell and synergids (the egg apparatus), as well as the early developing embryos from the zygote stage to the eight-cell stage [30]. If the activity of these two promoters in tobacco was consistent with what was observed in *Arabidopsis*, such temporal and spatial differences in the *PsASGR-BBML* expression likely resulted in more haploid progeny recovered in the RKD2gBB line than the ddBR1 lines, which was comparable to the observation where egg ablation by using the *barstar* gene happened more frequently when the gene was regulated by the *AtRKD2_pro_* than the *AtDD45_pro_* [30]. While RKD transcription factors play an essential role in maintaining the quiescent state of egg cells [31], expression of the *PsASGR-BBML* transgene in a temporal and spatial manner similar to the endogenous *RKD* genes appeared to relax the quiescent state of egg cells and promote parthenogenesis (inducing embryogenesis from unfertilized egg cells).

In addition to the differences of the promoter activity, it could be argued that the genome duplication in line RKD2gBB_6.1 might contribute to the higher efficiency of haploid production. Since the 4*x* T1 progeny of line RKD2gBB_6.1 still produced more haploid progeny than the ddBR1 lines (Table 1 and Table 2) and two other octoploid (8*x*) RKD2gBB lines, line RKD2gBB_4.1 and line RKD2gBB_7.1, recovered in this study did not produce any haploid progeny or at a very low frequency, respectively, the increase in ploidy would not be the only cause of more haploid production. We are unable to exclude the possibility that the particular integrations of the transgene in the genome could magnify the transgene effect in line RKD2gBB_6.1 which would require further investigation. Regardless of a similar frequency of haploid-producing lines identified, none of the ddBR1 lines reached the same level of haploid production as line RKD2gBB_6.1.

The efficiency of haploid production varied among lines, though a full-length of the *PsASGR-BBML* transgene was present in all these lines. Such variation was also observed in the previous study of rice and maize [10], probably due to positional differences in integration of the transgene. The transgenes seemed to segregate as a single locus in all haploid-producing lines except for line RKD2gBB_6.1 that had the transgenes integrated at two independent loci (Table S3). Based on the haploid production in the limited number of RKD2gBB_6.1 T1 progeny tested, the transgene at one locus was sufficient to induce haploid production (Table 2 and Table S4). A segregating population would be needed to determine whether both loci were functional and whether an additive effect of the two loci might lead to a higher frequency of haploid production. The segregating population could also help to unravel potentially unknown changes in the genome if any occurred that contributed to haploid production. Characterizing the integration sites of the transgene would be helpful to examine whether the transgene integration interrupted any genes associated with reproduction which might have enhanced the transgene effect on parthenogenesis. 

Homozygous progeny have not been recovered for any haploid-producing lines, except for one T1 plant (K6.1_7) of line RKD2gBB_6.1 that appeared to be a homozygote based on the qPCR data but was unable to set seeds due to the severe malformation of flowers. Previous study in rice and maize also had difficulty in recovering plants homozygous for the *PsASGR-BBML* transgenes [10]. Homozygosity potentially brought about embryo lethality given the segregation ratio of the T1 progeny derived from some haploid-producing lines (i.e., a 2:1 ratio in Table S3). The lack of fertile homozygous plants made it difficult to address the question of why not all egg cells that inherited the transgene showed the parthenogenesis phenotype (penetrance). The inflated numbers of non-transgenic progeny and a large proportion of seeds that failed to germinate in some T0 and T1 plants (Tables S3 and S4) suggested that the transgene or its integration site might affect embryo development and survival, which was observed in rice carrying the *PsASGR-BBML* transgenes [10].

Unlike sexual pearl millet carrying the *PsASGR-BBML* transgene where parthenogenetic embryos were observed in ovules of emasculated flowers without pollination [8], developing embryos were not observed in ovules of the haploid-producing transgenic tobacco lines unless emasculated flowers were hand-pollinated. Pseudogamous parthenogenesis observed in the *PsASGR-BBML* transgenic tobacco might be similar to what was observed in *Boechera* apomicts where self-pollination is still required to initiate the development of apomictic embryos as in sexual reproduction but the egg cell does not fuse with either sperm cell [32]. Since parthenogenetic progeny were obtained as long as the 4*x* progeny of line RKD2gBB_6.1 were pollinated regardless of whether the pollen carried the *PsASGR-BBML* transgene or not (Table 2 and Table 3), pollination could simply lead to fertilization of the central cell and endosperm formation to support development of parthenogenetic embryos into viable seeds, which was also observed and required in monocot species [8,10]. Fertilization of the central cells could generate certain critical signals to regulate early embryo development as observed in *Arabidopsis* [33] that would affect embryo survival in tobacco. Pollination also could deliver some paternal signals [34,35] that potentially influence parthenogenetic egg or zygote development. If parthenogenetic embryos began to develop without pollination, the slowness of embryo development in tobacco made it difficult to observe them, as most embryos only completed the first division in ovules seven days after pollination whereas flowers without pollination usually abscised six days after emasculation, as previously reported [27,36]. Taking advantage of a homozygous tobacco line carrying a *GmEF1a:DsRed:NOS* cassette as a pollen donor enabled us to identify parthenogenetic embryos that could be distinguished from the sexually reproducing embryos due to their lack of the paternal trait DsRed fluorescence. The haploidy characteristic of the DsRed negative seedlings was verified, indicating that the DsRed negative embryos did result from parthenogenesis.

## 5. Conclusions

Despite its origin from a monocot species, the *PsASGR-BBML* gene was shown to be functional in a dicot species, inducing haploid production in tobacco. Tuning the *PsASGR-BBML* transgene by altering the promoter seemed to improve the efficiency of haploid production. Compared with the monocot species previously studied, the lower penetrance of the gene in the dicot species tobacco probably stems from the divergence in the reproductive process and embryogenesis between monocots and dicots. Further investigation on the genetic network and protein interactions with the PsASGR-BBML transcription factor in egg cells may reveal the absence of monocot-specific co-factors or the presence of suppressors in dicots that hinder the full potential of the *PsASGR-BBML* gene to promote parthenogenesis.

## Figures and Tables

**Table 1:** Efficiency of haploid production in the haploid-producing T0 after self-pollination.

T0 line ID	Ploidy	# Total Seedlings	# Seedlings Screened ^1^	# Haploid T1 (%)
ddBR1_13.1	4*x*	1065	186	0 (0)
ddBR1_204.1	4*x*	785	101	0 (0)
ddBR1_204.2	4*x*	585	153	4 (0.7%)
ddBR1_205.1	4*x*	582	68	1 (0.2%)
ddBR1_240.2	4*x*	575	123	5 (0.9%)
RKD2gBB_3.2	4*x*	127	80	0 (0)
RKD2gBB_6.1	8*x*	129	117	12 (9.3%)
RKD2gBB_7.1	8*x*	223	65	0 (0)

^1^ For the ddBR1 lines, the seedlings screened were from a sub-population of seedlings which did not elongate when germinated on 0MS medium containing 60 mg/L hygromycin and remained shorter than 0.5 cm within 10 days.

**Table 2:** Efficiency of haploid production in the RKD2gBB_6.1 T1 progeny after self-pollination.

T1 Plant ID	Ploidy	# Total Seedlings	# Green Resistant Seedlings	# Seedlings Screened	# Haploid T2 (%)
K6.1_11	4*x*	103	57	56	13 (12.6%)
K6.1_14 ^1^	4*x*	-	-	-	-
K6.1_20	4*x*	387	111	101	14 (3.6%)
K6.1_23	4*x*	211	80	52	12 (5.7%)
K6.1_1	8*x*	51	40	40	8 (15.7%)
K6.1_7 ^1^	8*x*	-	-	-	-
K6.1_12	8*x*	224	173	150	50 (22.3%)
K6.1_17	8*x*	22	21	20	6 (27.3%)
K6.1_22	8*x*	75	73	60	2 (2.7%)

^1^ No flowers with pollen produced during the growing season, resulting in no progeny.

**Table 3:** Haploid production from a 4*x* RKD2gBB_6.1 T1 plant pollinated with wildtype pollen.

♀	♂	# Total Seedlings	# Green Resistant Seedlings	# Seedlings Screened	# Haploid Progeny (%)
K6.1_20	wildtype	285	71	56	16 (5.6%)

**Table 4:** Expression of DsRed in developing embryos of the crosses between 4*x* T2 plants of line RKD2gBB_6.1 and homozygous transgenic tobacco carrying a DsRed cassette.

Maternal Parent	# Crosses Investigated	# Total Embryos Observed	# DsRed + Embryos	# DsRed − Embryos	% Parthenogenetic (DsRed − Embryos)
wildtype	9	1497	1497	0	0
K6.1_1_35	4	446	428	18	4.0%
K6.1_12_26	8	537	504	33	6.1%
K6.1_12_75	1	250	242	8	3.2%
K6.1_12_104	1	155	150	5	3.2%
K6.1_17_7	6	550	506	44	8.0%

**Table 5:** Germination of progeny from the crosses between 4*x* T2 plants of line RKD2gBB_6.1 and homozygous transgenic tobacco carrying a DsRed cassette.

Maternal Parent	# Total Seedlings	# DsRed + Seedlings	# DsRed − Seedlings	# DsRed − Seedlings with Reduced Ploidy (Parthenogenesis %)
wildtype	220	220	0	0
K6.1_1_35	216	212	4	4 (1.8%)
K6.1_12_35	23	22	1	1 (4.3%)
K6.1_12_26	152	148	4	4 (2.6%)

## Supplementary Materials

The following are available online at https://www.mdpi.com/2073-4425/11/9/1072/s1, Figure S1: Flow cytometric seed screen (FCSS) analysis identified T0 plants that produced haploid progeny; Figure S2: Expression of the PsASGR-BBML transgene in the ovules from the haploid-producing 4*x* T1 of RKD2gBB_6.1 compared with non-haploid-producing RKD2gBB T0 lines; Table S1: Sequences of primers used for PCR; Table S2: T0 lines randomly selected to verify expression of the PsASGR-BBML transgene in ovules of flowers at developmental stage 11 by RT-PCR; Table S3. Genetic segregation and copy number estimate of the transgenes in the haploid-producing T0 lines; Table S4. Genetic segregation and copy number estimate of the transgenes in T1 progeny of line RKD2gBB_6.1; Table S5. Genetic segregation in progeny of a cross between a 4*x* RKD2gBB_6.1 T1 and non-transgenic tobacco.
