# Supplementary material for: Haploidy in Tobacco Induced by PsASGR-BBML Transgenes via Parthenogenesis
Source: Genes (Basel). 2020 Sep 12;11(9):1072. doi: 10.3390/genes11091072 (PMC7564442; doi:10.3390/genes11091072)
Supplement: Supplementary file 1 [file genes-11-01072-s001.pdf]

Haploidy in tobacco induced by *PsASGR-BBML* transgenes via parthenogenesis

Zhifen Zhang, Joann Conner, Yinping Guo and Peggy Ozias-Akins

Supplementary Materials:

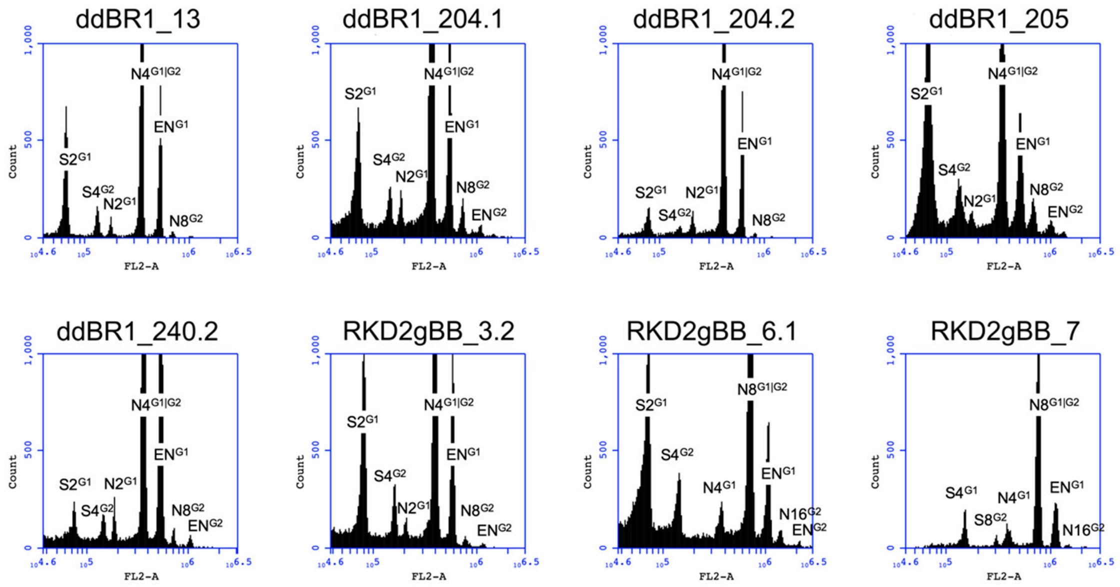

Figure S1. Flow cytometric seed screen (FCSS) analysis identified T0 plants that produced haploid progeny. $S2^{G1}$, $S4^{G2}$, $S4^{G1}$, and $S8^{G2}$ designates 2n/2x/2c, 2n/2x/4c, 2n/4x/4c, and 2n/4x/8c peaks of sorghum, respectively; $N2^{G1}$, $N4^{G1}$, $N4^{G1|G2}$, $N8^{G2}$, $N8^{G1|G2}$, and $N16^{G2}$ designate 2n/2x/2c, 2n/4x/4c, 2n/4x/4c|2n/2x/4c, 2n/4x/8c, 2n/8x/8c|2n/4x/8c, and 2n/8x/16c peaks of tobacco embryos, respectively; EN^{G1} and EN^{G2} designate the G1 and G2 peaks of endosperm from tobacco seeds

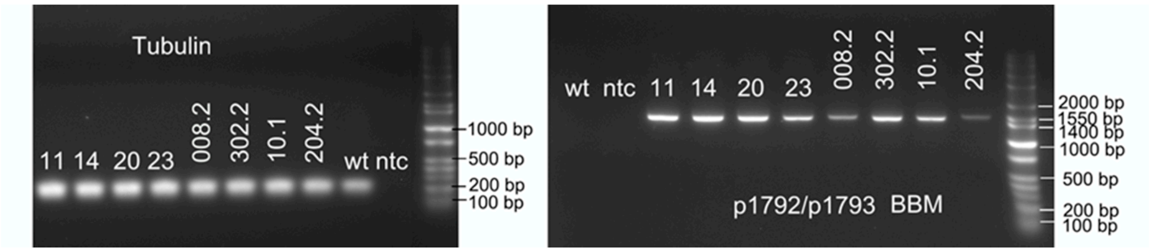

Figure S2. Expression of the *PsASGR-BBML* transgene in the ovules from the haploid-producing 4x T1 of RKD2gBB_6.1 compared with non-haploid-producing RKD2gBB T0 lines. Left: RT-PCR with p4133/4134 for the *NtTubulin* gene (XM_016623993) as a reference gene; right: RT-PCR with p1792/1793 showing the *PsASGR-BBML* transcript including 8 exons. 11-23: 4x T1 of line RKD2gBB_6.1, K6.1_11, K6.1_14, K6.1_20, and K6.1_23, respectively; 008.2, 302.2, 10.1 and 204.2: non-haploid-producing RKD2gBB T0 lines, RKD2gBB_8.2, RKD2gBB_302.2, RKD2gBB_10.1, RKD2gBB_204.2, respectively; wt: non-transgenic tobacco; ntc: no template PCR

Table S1. Sequences of primers used for PCR

primers	description	sequence 5’ to 3’	annealing temperature (°C)
p3905	*AtRKD2* promoter forward	ACTTCATTAATAACTTATGATTAATTATG	55
p3906	*AtRKD2* promoter reverse	ATCTTAAGGAAAGTGAGAGAGATA	55
p1792	*PsASGR-BBML* full-length forward	TTCCACCAACAACTGGCTGCGCT	60
p1801	*PsASGR-BBML* full-length reverse	TTCTCATGGCTCCTAGACTCCCAC	60
p1793	*PsASGR-BBML* exon 8 reverse	CACCATGGTAACCAACGTCCCC	60
p4303	qPCR, *PsASGR-BBML* forward	GCTTCATACCTTCCTGTCCATC	60
p4304	qPCR, *PsASGR-BBML* reverse	TCCCTTCGTGCCTGTTTATC	60
p4127	*nptII* forward	CCTGCCGAGAAAGTATCCATC	60
p3767	*nptII* reverse	AAAAGCGGCCATTTTCCACC	60
p4133	*NtTubulin* forward	GTACATGGCCTGTTGTTTGATG	60
p4134	*NtTubulin* reverse	CTGGATGGTCCTCTTTGTCTTT	60
p3702	*DsRed* forward	GGCTCCAAGGTGTACGTGAA	60
p3703	*DsRed* reverse	GGTGTAGTCCTCGTTGTGGG	60

Table S2. T0 lines randomly selected to verify expression of the *PsASGR-BBML* transgene in ovules of flowers at developmental stage 11 by RT-PCR

construct	# lines tested	plant ID[1] with the *PsASGR-BBML* transcript including 8 exons verified
ddBR1 (*AtDD45:gASGR-BBML*)	10	1.1, 8.1, 10.1, 11.1, **204.2**, **205.1**, 12.7, 215.2, 217.1, 240.3
RKD2gBB (*AtRKD2:gASGR-BBML*)	10	1.2, 2.2, 4.1, 5.2, **6.1**, 10.1, 10.2, 204.2, **7.1**, 8.2, 302.2

[1] The plant ID in bold indicates the plants with haploid signal detected by FCSS

Table S3. Genetic segregation and copy number estimate of the transgenes in the haploid-producing T0 lines

T0 line ID	# T1 resistant to antibiotic (R)	# T1 susceptible to antibiotic (S)	# seeds not germinated	segregation ratio R:S (p-value)	copy# estimated by qPCR (BBM/Tubulin)
ddBR1_13.1	691	374	112	2:1 (0.22)	2 (0.87)
ddBR1_204.1	439	346	190	1:1 (0.001)	1 or 2 (0.59)
ddBR1_204.2	424	161	0	3:1 (0.16)	2 or 3 (1.28)
ddBR1_205.1	472	110	26	3:1 (0.001)	2 (0.91)
ddBR1_240.2	392	183	264	2:1 (0.44)	3 or 4 (1.79)
RKD2gBB_3.2	99	28	260	3:1 (0.44)	-
RKD2gBB_6.1	120	9	250	15:1 (0.73)	2 (0.66)
RKD2gBB_7.1	141	82	302	2:1 (0.28)	2 (1.08)

Table S4. Genetic segregation and copy number estimate of the transgenes in T1 progeny of line RKD2gBB_6.1

T1 plant ID	ploidy	# resistant T2 seedlings (R)	# susceptible T2 seedlings (S)	# seeds not germinated	segregation ratio R:S (p-value)	# copy estimated by qPCR (BBM/Tubulin)
K6.1_11	4x	57	46	84	1:1 (0.28)	2 (1.03)
K6.1_14[1]	4x	-	-	-	-	2 or 3 (1.31)
K6.1_20	4x	111	276	37	1:3 (0.09)	3 or 4 (1.70)
K6.1_23	4x	80	131	7	1:2 (0.16)	3 (1.46)
K6.1_1	8x	40	11	53	3:1 (0.57)	1 or 2 (0.72)
K6.1_7[1]	8x	-	-	-	-	4 (2.27)
K6.1_12	8x	173	51	2	3:1 (0.44)	1 (0.52)
K6.1_17	8x	21	1	20	15:1 (0.74)	2 (1.00)
K6.1_22	8x	73	2	117	15:1 (0.20)	1 (0.28)

[1] No flowers with pollen produced during the growth season, resulting in no progeny

Table S5. Genetic segregation in progeny of a cross between a 4x RKD2gBB_6.1 T1 and non-transgenic tobacco

♀	♂	# green/ resistant seedlings (R)	# white/ susceptible seedlings (S)	# total seedlings	# seeds not germinated	segregation ratio R:S (p-value)
K6.1_20	wild type	71	214	285	95	1:3 (0.97)
